# A Fe^2+^-dependent self-inhibited state influences the druggability of human collagen lysyl hydroxylase (LH/PLOD) enzymes

**DOI:** 10.3389/fmolb.2022.876352

**Published:** 2022-08-25

**Authors:** Luigi Scietti, Elisabetta Moroni, Daiana Mattoteia, Marco Fumagalli, Matteo De Marco, Lisa Negro, Antonella Chiapparino, Stefano A. Serapian, Francesca De Giorgi, Silvia Faravelli, Giorgio Colombo, Federico Forneris

**Affiliations:** ^1^ The Armenise-Harvard Laboratory of Structural Biology, Department of Biology and Biotechnology, University of Pavia, Pavia, Italy; ^2^ Consiglio Nazionale delle Ricerche, Istituto di Scienze e Tecnologie Chimiche “Giulio Natta” (SCITEC-CNR), Milano, Italy; ^3^ Department of Chemistry, University of Pavia, Pavia, Italy

**Keywords:** collagen, lysyl hydroxylase (LH), Fe2+/2-oxoglutarate-dependent dioxygenases, structure-based drug design, molecular dynamics simulations, cancer metastasis, 2-2'-bipyridyl

## Abstract

Multifunctional human collagen lysyl hydroxylase (LH/PLOD) enzymes catalyze post-translational hydroxylation and subsequent glycosylation of collagens, enabling their maturation and supramolecular organization in the extracellular matrix (ECM). Recently, the overexpression of LH/PLODs in the tumor microenvironment results in abnormal accumulation of these collagen post-translational modifications, which has been correlated with increased metastatic progression of a wide variety of solid tumors. These observations make LH/PLODs excellent candidates for prospective treatment of aggressive cancers. The recent years have witnessed significant research efforts to facilitate drug discovery on LH/PLODs, including molecular structure characterizations and development of reliable high-throughput enzymatic assays. Using a combination of biochemistry and *in silico* studies, we characterized the dual role of Fe^2+^ as simultaneous cofactor and inhibitor of lysyl hydroxylase activity and studied the effect of a promiscuous Fe^2+^ chelating agent, 2,2’-bipyridil, broadly considered a lysyl hydroxylase inhibitor. We found that at low concentrations, 2,2’-bipyridil unexpectedly enhances the LH enzymatic activity by reducing the inhibitory effect of excess Fe^2+^. Together, our results show a fine balance between Fe^2+^-dependent enzymatic activity and Fe^2+^-induced self-inhibited states, highlighting exquisite differences between LH/PLODs and related Fe^2+^, 2-oxoglutarate dioxygenases and suggesting that conventional structure-based approaches may not be suited for successful inhibitor development. These insights address outstanding questions regarding druggability of LH/PLOD lysyl hydroxylase catalytic site and provide a solid ground for upcoming drug discovery and screening campaigns.

## Introduction

**FIGURE 1:**
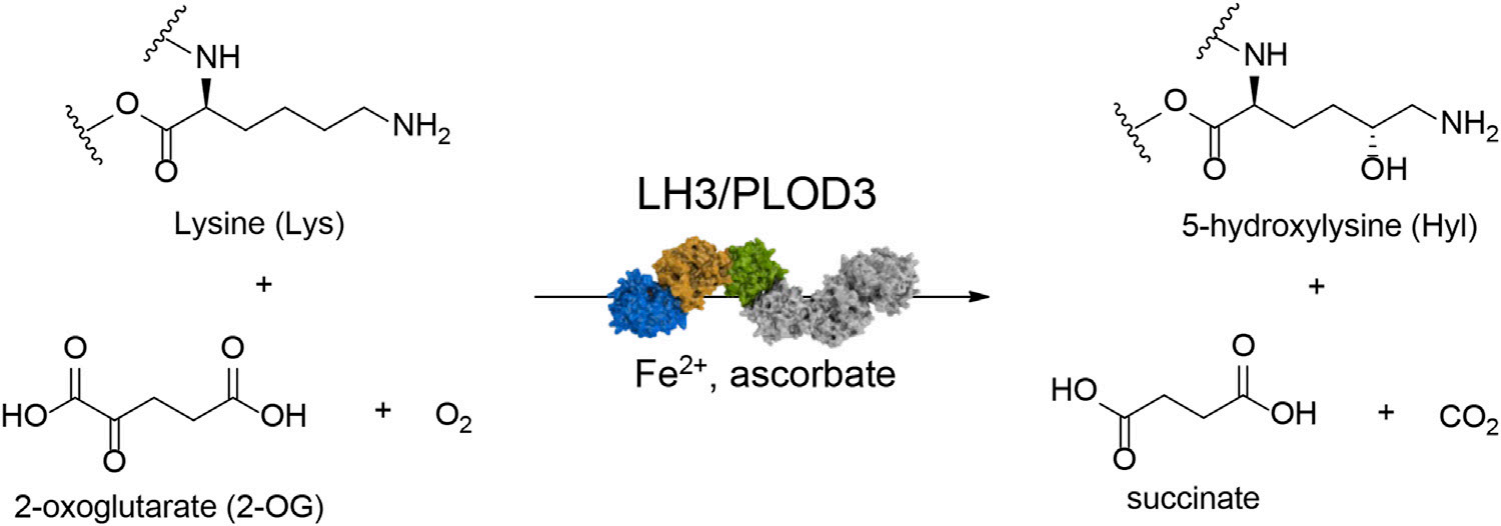
Reaction scheme for collagen lysine hydroxylation by LH3/PLOD3. The enzyme requires Fe^2+^ as an essential catalytic cofactor, O_2_, and 2-oxoglutarate (2-OG) as co-substrates, and ascorbate to maintain the redox state of Fe^2+^ to enable catalytic turnover. Upon lysine hydroxylation, 2-OG is converted into succinate with release of one molecule of CO_2_.

The supramolecular organization of collagen in the extracellular matrix (ECM) depends on various post-translational modifications (PTMs) that occur during its biosynthesis. Among the different PTMs, lysine (Lys) hydroxylation is key for proper collagen fibril formation, thus defining the overall physiochemical properties of ECM (Yamauchi and Sricholpech, 2012). The collagen lysyl hydroxylase (LH/PLOD) enzyme family comprises the three isoforms LH1/PLOD1, LH2/PLOD2 and LH3/PLOD3 (encoded by the procollagen-lysine, 2-oxoglutarate 5-dioxygenase (*PLOD*) genes) and is the sole enzyme capable of hydroxylating collagen Lys in humans (Scietti and Forneris, 2020). These enzymes use Fe^2+^, 2-oxoglutarate (2-OG), ascorbate and O_2_ to catalyze the addition of a hydroxyl group in position 5 of collagen Lys, yielding 5-hydroxylysine (Hyl) with the release of succinate and CO_2_ (Figure 1). Unmodified collagen Lys and modified Hyl are both substrates of collagen lysyl oxidases (LOX), which catalyze the oxidative deamination of Lys and Hyl forming highly reactive aldehydes (Lys^ald^ and Hyl^ald^, respectively) that spontaneously rearrange to form Lys-derived collagen cross-links (LCC) and Hyl-derived collagen cross-links (HLCC) in the ECM. A physiological ratio between LCC and HLCC is essential to establish and maintain a proper ECM functionality. Conversely, excess HLCC in the tumor microenvironment has been linked to biomechanical alterations and increased ECM tension and stiffness (Levental et al., 2009; Pankova et al., 2016). The deposition of ordered thicker collagen fibers, as consequence of the abnormal LCC/HLCC ratio, is a characteristic of severe tissue fibrosis, one of the hallmarks of cancer (Van Der Slot et al., 2004; Chen et al., 2015). Cancer cells take advantage of these “collagen highways” to migrate toward blood vessels that sustain metastatic progression (Provenzano et al., 2006; Du et al., 2017b; Gkretsi and Stylianopoulos, 2018). Over the last years, multiple studies correlated both hypoxia-dependent and independent overexpression and mislocalization of LH enzymes with increased propensity to metastatization in a wide variety of solid tumors, recognizing these enzymes as markers of adverse prognosis (Chen et al., 2015; Chen et al., 2016; Pankova et al., 2016; Sato et al., 2021).

Initially, the hypoxia-driven overexpression of *PLOD2* was the first identified prognostic factor in several tumors, as hepatocellular carcinoma (Noda et al., 2012; Du et al., 2017a), sarcoma (Eisinger-Mathason et al., 2013), lung and colon cancer (Du et al., 2017a), renal carcinoma (Kurozumi et al., 2016), glioma (Song et al., 2017; Xu et al., 2017), oral squamous cell and endometrial carcinoma (Saito et al., 2019; Wan et al., 2020) bone and breast metastasis (Blanco et al., 2012; Gilkes et al., 2013; Du et al., 2017b) and cervical cancer (Li et al., 2021). Interestingly, the downregulation of LH2/PLOD2 isoform in renal cell carcinoma via tumor suppressing miRNA significantly inhibited cell migration and invasion (Kurozumi et al., 2016), confirming the importance of LH in tumor progression. Later on, the LH1/PLOD1 and the LH3/PLOD3 isoforms were also identified as biomarkers in many different types of solid tumors. High *PLOD1* expression levels were found in gastrointestinal carcinoma (Wang et al., 2018), osteosarcoma (Jiang et al., 2020), glioma (Tian et al., 2021; Wang et al., 2021) and bladder cancer (Yamada et al., 2019). The LH3/PLOD3 isoform was identified to be upregulated in glioma (Tsai et al., 2018; Baek et al., 2019), gastric cancer (Wang et al., 2019) and colorectal cancer (Deng et al., 2021; Shi et al., 2021), acting as a promoter of metastatization in different cancer types (Gong et al., 2021). In agreement with the observations on *PLOD2*, also *PLOD3* knockdown suppressed the malignant phenotype in renal cell carcinoma (Xie et al., 2020). Furthermore, the entire LH/PLOD family was found correlated with metastatization of solid tumors as hepatocellular and renal cell carcinomas (Xu et al., 2019; Yang et al., 2020), gliomas (Zhao et al., 2021), gastric (Li et al., 2020), and ovarian (Guo T. et al., 2021) cancers. Taken together, these observations strongly point these enzymes as a hot topic in cancer research: LH/PLODs are not only widely recognized prognostic markers of cancer metastatization with poor outcome, but also very promising druggable targets for anticancer therapy.

The lack of a structural templates of LH/PLODs have hampered for many years targeted drug discovery campaigns. Only recently, our group and others determined molecular structures suitable as templates for *in silico* drug discovery (Guo et al., 2018; Scietti et al., 2018). In particular, the crystal structure of the full-length LH3/PLOD3, the first of a human collagen lysyl hydroxylase, provided key insights on catalytic pockets. LH3/PLOD3 is a multifunctional enzyme capable of performing collagen lysine hydroxylation (as the other two isoforms) and glycosylation (Scietti and Forneris, 2020; De Giorgi et al., 2021). Indeed, LH3/PLOD3 can additionally catalyze the galactosylation and further glucosylation of Hyl to form α-(1,2)-glucosyl-β-(1,O)-galactosyl-5-hydroxylysine *in vitro*. The identification of specific genes (*COLGALT1/*2) encoding for collagen galactosyltrasferases (GLT25D1/2) makes the physiological relevance of the galactosyltransferase activity of LH3/PLOD3 under debate. In addition, there are increasing indications that also LH1/PLOD1 and LH2/PLOD2 possess glycosyltransferase activity, although less pronounced (Ewans et al., 2019; Guo H. F. et al., 2021).

**FIGURE 2:**
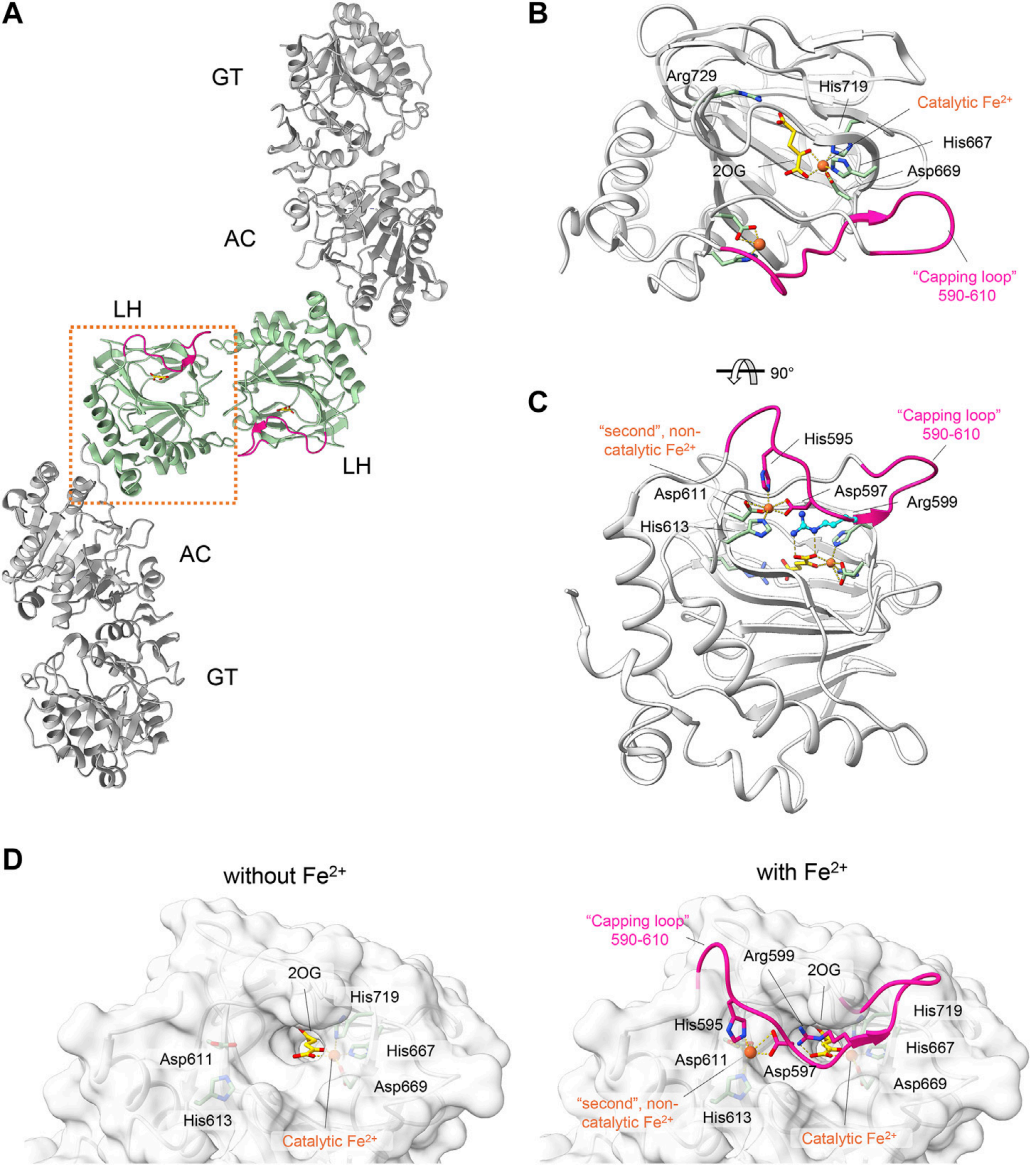
Structural features of the LH domain and catalytic site in LH3/PLOD3. **(A)** Cartoon representation of the dimeric assembly of multidomain LH3/PLOD3. The labels indicate the domain organization of LH3/PLOD3, showing the positions of the GT, AC, and LH domains along the quaternary structure. The box highlights the position of the LH domain (green) at the dimer interface. **(B)** Details of the LH catalytic site. Amino acids surrounding the Fe^2+^ cofactor and 2-OG are shown as sticks. The capping loop is shown in magenta, indicating the substrate entrance site. **(C)** Details of the second Fe^2+^ binding site interlocking the capping loop into the self-inhibitory state. Residues interacting with the second Fe^2+^ as shown as sticks. Residue Arg599, mimicking the collagen lysine substrate subject to hydroxylation, is shown in blue. **(D)** Side-by-side surface representations of the accessibility to the LH active site in absence (left) and in presence (right) of the capping loop. Residues involved in catalysis and in maintaining the self-inhibitory state of the capping loop are shown as in panels **(B,C)**.

LH/PLOD enzymes belong to the Fe^2+^, 2-OG-dependent dioxygenase superfamily (Martinez and Hausinger, 2015), a widespread class of enzymes that catalyzes oxidative reactions such as epimerization, demethylation, and hydroxylation (Hausinger, 2004; Flashman and Schofield, 2007; Loenarz and Schofield, 2008; Loenarz and Schofield, 2011). Despite the broad range of functions carried out, all Fe^2+^, 2-OG-dependent dioxygenases display a common double-stranded β-helix folding (DSBH) topology with highly conserved binding sites and catalytic mechanisms (Costas et al., 2004; Clifton et al., 2006). The catalytic domain responsible for Lys hydroxylation is located at the C-terminus of the LH/PLOD structure and is essential for its unique dimeric quaternary structure, a fundamental prerequisite for collagen lysyl hydroxylase activity (Guo et al., 2018; Scietti et al., 2018). Within the enzymatic pocket of human LH3/PLOD3, a catalytic Fe^2+^ is coordinated by His667, Asp669 and His719 (Figures 2A,B). In a LH/PLOD viral homolog, this Fe^2+^ is a fundamental structural element of LH/PLOD enzymes and its chelation from the active site completely disrupt protein folding, dimer formation and catalytic activity (Guo et al., 2018). Pioneering work (Kivirikko and Prockop, 1967; Myllylä et al., 1979; Puistola et al., 1980) highlighted the complexity and binding promiscuity of LH/PLODs towards binding of different metal ions, and the associated impact on the LH enzymatic activity. Structural studies also revealed the presence of a possible second Fe^2+^ bound within the LH domain, shaping a unique site never observed in other Fe^2+^, 2-OG-dependent dioxygenases. This second Fe^2+^ is coordinated by two Asp and two His residues (in human LH3/PLOD3, His595, Asp597, Asp611 and His613), whose side chain interactions with the metal ion induce a well-defined conformation of a “capping loop”, a stretch comprising residues Gly590-Glu610 that closes the entrance of the catalytic site, mimicking the collagen Lys substrate by positioning Arg599 exactly in front of the 2-OG donor substrate (Scietti et al., 2018) (Figures 2C,D). The same region was characterized by pronounced flexibility in absence of Fe^2+^ (Guo et al., 2018; Scietti et al., 2018), but did not allow to unambiguously rule out crystallization-induced stabilization of the unique conformation observed in the presence of a second Fe^2+^ bound.

The simultaneous presence of features common to all Fe^2+^, 2-OG-dependent dioxygenases and unique, distinguishing elements exclusively present in the LH/PLOD family, makes these enzymes ideal targets for structure-based drug discovery campaigns. In this study, we combined MD simulations with structure-guided mutagenesis of LH3/PLOD3 and used biochemical assays to elucidate the role of the capping loop in the accessibility of the active site. Our work sets the grounds for successful drug discovery campaigns on LH/PLOD enzymes to fight cancer metastasis.

## Materials and methods

### Chemicals

All chemicals were purchased from Sigma-Aldrich (Merck) unless specified otherwise.

### Molecular cloning and site-directed mutagenesis

The coding sequence for wild-type human *PLOD3* gene (GenBank accession number BC011674.2) was obtained from Source Bioscience. Oligonucleotides containing in-frame 5′-BamHI and 3′-NotI were designed and used to sub-clone the coding sequence devoid of the N-terminal signal peptide into a pCR8 vector, that was also used as a template for subsequent experiments. Single-point mutations were generated using Phusion Site Directed Mutagenesis (Invitrogen) with the oligonucleotides listed in Table 1. The linear mutagenized plasmids were phosphorylated using T4 polynucleotide kinase (Invitrogen) prior to ligation using T4 DNA ligase (Invitrogen). All plasmids were checked by Sanger sequencing prior to cloning into the pUPE.106.08 expression vector. This expression vector, kindly provided by U-protein Express, BV (U-PE, Netherlands) provides the N-terminal cystatin signal peptide, followed by a N-terminal 6xHis-tag and a recognition site for Tobacco Etch Virus (TEV) protease prior to the in-frame BamHI-NotI restriction cassette, followed by an in-frame stop codon.

**TABLE 1:** List of oligonucleotides used to generate LH3/PLOD3 mutants.

Oligonucleotide name	Oligonucleotide sequence
D597A-Fw	CTT​CAA​GGC​TGG​CTG​GAG​GCT​AC
D597A-Rv	CCT​CAT​GCC​GGC​CGC​CTG​AC
D611A-Fw	CCA​TCC​ACA​TGA​AGC​AGG​TGG​GG
D611A-Rv	CCA​CGG​TGG​GCA​CAT​TCT​CGT​AG

### Production of recombinant LH3/PLOD3 expression using transiently-transfected HEK293F cells

Recombinant tagged LH3/PLOD3 were produced using suspension cultures of HEK293F (Invitrogen) cells, maintained and transfected according to (Faravelli et al., 2021). Cells were not authenticated and not tested for *mycoplasma* contamination. Briefly, cells were transfected at cell densities of 1 million/ml using 3 μg of polyethyleneimine (PEI; Polysciences) for 1 μg of pUPE.106.08-LH3/PLOD3 plasmid DNA per mL of cells. Cultures were supplemented with 0.6% Primatone RL 4 h after transfection. The cell medium containing secreted LH3/PLOD3 was collected 6 days after transfection by centrifugation at 1,000 × g for 15 min.

### Purification of recombinant LH3/PLOD3 enzymes

The LH3/PLOD3-containing medium from HEK293F cell cultures was filtered through a syringe 0.8 μm filter (Sartorius). The pH and ionic strength of the filtered medium were adjusted using a 5X concentrated buffer stock to reach a final concentration of 25 mM 4-(2-hydroxyethyl)−1-piperazineethanesulfonic acid (HEPES)/NaOH, 500 mM NaCl, 30 mM imidazole, pH 8.0. Recombinant LH3/PLOD3 was purified using a combination of affinity and size-exclusion chromatography on Äkta systems (GE Healthcare) according to (Scietti et al., 2018). The filtered supernatant was first loaded onto a 20 ml His-Prep FF column (GE Healthcare) and eluted using 250 mM imidazole. The eluate was then loaded onto a 5 ml HiPrep desalting FF column (GE Healthcare) equilibrated in 25 mM HEPES/NaOH, 500 mM NaCl, pH 8.0. The N-terminal His-tag was cleaved using overnight His-tagged TEV protease digestion at 4°C followed by affinity-based removal of TEV protease and the cleaved His-tag using a 5 ml HisTrap FF (GE Healthcare). Recombinant LH3 was concentrated to 5 mg/ml using 30,000 MWCO Vivaspin Turbo centrifugal filters (Sartorius), then loaded onto a Superdex 200 10/300 GL (preparative scale) or onto a Superdex 200 5/150 GL (analytical scale) columns (GE Healthcare) equilibrated with 25 mM HEPES/NaOH, 200 mM NaCl, pH 8.0. LH3/PLOD3-containing fractions as assessed from SDS-PAGE analysis were pooled, concentrated, and stored at −80°C until further usage.

### LH assays using LC-MS and analysis of 2,2’-bipyridil effects on enzymatic activity

Synthetic collagen peptides were purchased from China Peptides. Peptides tested were ARGIKGIRGFS and GIKGIKGIKGIK sequences (Scietti et al., 2018). 5 μM LH3/PLOD3 was incubated with 50 μM FeCl_2_, 100 μM 2-OG, 500 μM ascorbate, 1 mM peptide substrate and 0–500 μM 2,2′-bipyridine. Reactions were allowed to proceed for 3 h at 37°C. 10 μl of each sample were supplemented with 38 μl of Milli-Q water and acidified by addition of 2 μl of formic acid (FA) to reach a final volume of 50 μl, then analyzed on an UHPLC-HRMS/MS system (AB Sciex, United States). LC unit (ExionLC AD) consists of a column oven thermostated at 40°C, an autosampler cooled at 10°C and a binary gradient pump system. MS instrument consists of a high resolution QTOF mass spectrometer (AB Sciex X500B) equipped with a Turbo V Ion source and a Twin Sprayer ESI (electrospray ionization) probe, controlled by SCIEX OS 2.1 software. Peptides were separated by reverse phase (RP) HPLC on a Hypersil Gold C18 column (150 × 2.1 mm, 3 μm particle size, 175 Å pore size, Thermo Fisher Scientific) using a linear gradient (2–50% solvent B in 15 min) in which solvent A consisted of 0.1% aqueous FA and solvent B of acetonitrile (CAN) containing 0.1% FA. Flow rate was 0.2 ml/min. Mass spectra were generated in positive polarity under constant instrumental conditions: ion spray voltage 4,500 V, declustering potential 100 V, curtain gas 30 psi, ion source gas 1 40 psi, ion source gas 2 45 psi, temperature 350°C, collision energy 10 V. Spectra analyses were performed using SCIEX OS 2.1 software. Statistical evaluations based on pair sample comparisons between uncoupled and coupled assay values using Student’s *t*-test in Prism 7 (Graphpad software).

### Luminescence-based LH assays

Reaction mixtures (5 μl total volume) were prepared according to (Scietti et al., 2018) by sequentially adding LH3/PLOD3 at 0.2 mg/ml, 0–1 mM peptide substrate or 4 mg/ml gelatin in water (solubilized through heating denaturation at 95°C for 10 min), 500 μM ascorbate, 100 μM 2-OG, variable concentrations of FeCl_2_ (0–200 μM) and let incubate for 1 h at 37°C. Reactions were stopped by heating samples at 95°C for 2 min prior to transfer into Proxiplate white 384-well plates (Perkin-Elmer), then 5 μl of the Succinate-Glo reagent I (Promega) were added and let incubate 1 h at 25°C, after that 10 μl of the Succinate-Glo reagent II (Promega) were added and let incubate 10 min at 25°C. The plates were then transferred into a GloMax Discovery plate reader (Promega) configured according to manufacturer’s instructions for luminescence detection. All experiments were performed in triplicates. Control experiments were performed using identical conditions by selectively removing LH3/PLOD3, 2-OG or peptide substrates. Data were analyzed and plotted using Prism 7 (Graphpad Software). Statistical evaluations based on pair sample comparisons between uncoupled and coupled assay values using Student’s t-test in Prism 7 (Graphpad software).

### Differential scanning fluorimetry assays

DSF assays were performed on LH3/PLOD3 wild-type using a Tycho NT.6 instrument (NanoTemper Technologies). LH3/PLOD3 samples at a concentration of 1 mg/ml in a buffer composed of 25 mM HEPES/NaOH, 200 mM NaCl, pH 8. Binding assays were performed by incubating LH3/PLOD3 with variable FeCl_2_ and 2,2′-bipyridil concentrations. Data were analyzed and plotted using GraphPad Prism 7 (Graphpad Software).

### Fe^2+^ binding assays

Recombinant LH3/PLOD3 was subject to labeling using the NHS-RED kit (NanoTemper Technologies) according to manufacturer’s instructions. Labeled LH3/PLOD3 at a concentration of 50 nM was incubated in a buffer composed of 25 mM TRIS/HCl, 100 mM NaCl, pH 7.5 with variable concentrations of FeCl_2_ for 40 min. The samples were then transferred into Dianthus 384-well plates for Temperature-Related Intensity Change (TRIC) (NanoTemper Technologies), and centrifuged at 1,000 g for 2 min. TRIC measurements were performed immediately after centrifugation using a Dianthus NT.23 instrument (NanoTemper Technologies). The samples were first measured for 1 s without heating and for 5 s with the IR-laser turned on. Normalized fluorescence values (*F*_*norm*_), described as ratios between fluorescence values after and prior to infrared laser activation were collected and plotted as a function of ligand concentration (Schulte et al., 2021). Determination of binding affinities was carried out using the DI.Screening Analysis software (NanoTemper Technologies). Data were then exported and plotted using GraphPad Prism 7 (Graphpad Software).

### Molecular dynamics simulations

Simulations were started from the extrapolation of the dimeric LH domain of human LH3/PLOD3 from its experimental crystal structure (PDB 6FXR) (Scietti et al., 2018) using *COOT* (Emsley et al., 2010); two systems were prepared: in one system both Fe^2+^ cations were left (LH3_Fe2_), while in the other one only the catalytic Fe^2+^ was left in (LH3_Fe1_). Residues were modeled in their standard protonation states at physiological pH, as predicted by *PROPKA*, version 3.1 (Sondergaard et al., 2011): this resulted in one disulfide bridge (between Cys 563 and 698), histidines 546, 586, 643, 681, 717 being protonated on Nε2, and histidines 595, 613, 667, 711, 719 protonated on Nδ1 (comprising histidines in both Fe^2+^ binding sites). Crystallographic waters were taken from the published crystal structure of LH3/PLOD3 (PDB: 6FXR) (Scietti et al., 2018). Hydrogen atoms were introduced using the *tleap* utility in *AmberTools* (version 19) (Case et al., 2005), as well as –NH_3_^+^ and –COO^–^ caps at the *N-* and *C-*termini, respectively. All molecular dynamics simulations (MD) were carried out with the *AMBER* software package (version 18) (Case et al., 2005; Case et al., 2018), using its GPU-accelerated (Salomon-Ferrer et al., 2013) *pmemd.cuda* utility during equilibration and production, and *sander* otherwise; three independent MD replicas (different random seeds) were carried out for LH3_Fe1_ and LH3_Fe2_ alike. A 8.0 Å cutoff was applied for the calculation of Lennard-Jones and Coulomb interactions between nonbonded atoms; beyond this limit, only Coulomb interactions were computed, using the particle mesh Ewald approach (Darden et al., 1993). Each replica’s production stage was 1 μs in length, and conducted with a 2 fs time-step in the *NpT* ensemble (with a temperature of 300 K enforced *via* Langevin’s thermostat (Loncharich et al., 1992); collision frequency 1 ps^–1^, and a 1 atm pressure enforced by Berendsen’s barostat) (Berendsen et al., 1984). Preproduction stages for each replica consisted in minimization (10 steps of steepest descent + 290 steps of conjugate gradient); heating (20 ps; *NpT*; 25–300 K; increasingly softer harmonic restraints on Cα atoms; *k* = 5.0 kcal mol^–1^ Å, collision frequency 0.75 ps^–1^ , with 2 fs time-step); and equilibration (1.0 ns; *NpT*; 300 K collision frequency 1 ps^–1^ , with 2 fs time-step). Analyses of MD trajectories were carried out with the *CPPTRAJ* program distributed within the *AmberTools* suite (version 19) (Case et al., 2005) or with code written in-house.

### Distance fluctuation

To characterize the impact of the second Fe^2+^ on the internal dynamics of LH domain of LH3, we made use of the previously introduced distance fluctuation (DF) analysis (Morra et al., 2012; Moroni et al., 2018). For each MD trajectory of the two systems, we computed on the combined meta trajectory the matrix of distance fluctuations, in which each element of the matrix corresponds to the DF parameter. DF is defined, for a couple of amino acids *i* and *j*, as the variance of the time-dependent distance *d*_*ij*_ of the C α atoms:

$$DF_{ij\;}=\langle (d_{ij\;}-\;\langle d_{ij\;}\rangle {)}^{2}\rangle$$

where the brackets indicate the time-average over the trajectory. This parameter is invariant under translations and rotations of the molecules and, unlike the covariance matrix, does not depend on the choice of a particular protein reference structure. The resulting DF matrix can be used to assess the intrinsic flexibility of proteins. This parameter characterizes residues that move in a highly coordinated fashion, and it is actually able to reflect the presence of specific coordination patterns and quasi-rigid domains motion in the protein of interest. In particular, pairs of amino acids belonging to the same quasi-rigid domain are associated with small distance fluctuations and vice versa.

### Forcefield and parametrization of cofactor 2-OG and Fe^2+^ binding sites

Lennard-Jones and intramolecular bonded parameters for the 2-oxoglutaric acid cofactor (2-OG) in the catalytic site of LH3/PLOD3 were assigned according to the *generalized Amber forcefield* (GAFF) (Wang et al., 2004), using *AmberTools’ antechamber* and *parmchk2* utilities (Case et al., 2005), after adding methyl hydrogens using the *reduce* tool (Case et al., 2005). Assignment of charges and (intermolecular) 2-OG-Fe^2+^ bonded parameters were performed as discussed below. Parametrization of both Fe^2+^ binding sites was carried out using the *MCPB.py* utility (Li and Merz, 2016) in conjunction with density functional theory calculations (DFT) using the *Gaussian09* suite (Frisch et al., 2009). Intermolecular bonded parameters for all residues in both Fe^2+^-binding sites and for the 2-OG cofactor were derived by *MCPB.py* (Li and Merz, 2016) applying the Seminario method (Seminario, 1996) on a DFT-derived Hessian matrix. More specifically, this Hessian matrix was calculated at the B3LYP (Lee et al., 1988; Becke, 1993)/6-31G(*d*) level of theory after optimizing a “small” model of both binding sites at the same level to a confirmed minimum (no imaginary frequencies). Such “small” models (generated by *MCPB.py* (Li and Merz, 2016)) included: 1) the Fe^2+^ cation in that particular binding site; 2) binding site residue sidechains up to their Cβ, with a shorter Cβ-H bond replacing Cα-Cβ; and 3) in the catalytic site, the entire 2-OG cofactor. Residues’ Cβ atoms and 2-OG’s carboxylate oxygens farthest from Fe^2+^ were frozen during optimization and excluded from frequency calculations. Atomic point charges on both Fe^2+^ cations on individual binding site residues and on the entire 2-OG cofactor were fitted by *MCPB.py* (Li and Merz, 2016) using the *RESP* method (Bayly et al., 1993) based on the outcome of (single-point) *ESP* charge fitting calculations (Besler et al., 1990) at a higher DFT level, on a “large” version of each Fe^2+^ binding site. More specifically, the chosen level of DFT for *ESP* charge fitting (Besler et al., 1990) was B3LYP (Lee et al., 1988; Becke, 1993)/6-31G(*d*)/def2-SV(P), (Weigend and Ahlrichs, 2005) with the def2-SV(P) basis set specifically applied to Fe^2+^. “Large” binding site models (generated by *MCPB.py* (Li and Merz, 2016)) comprised Fe^2+^, 2-OG, binding site residues in their entirety (i.e., with their backbone) as well as backbones of Glu596 and His668, contiguous to His595/Asp597 and His667/Asp669, respectively. All contiguous backbone fragments in the large models are capped by acetyl and *N-*methyl moieties at their *N-* and *C-*termini, respectively. *Gaussian09* (Frisch et al., 2009) was programmed to perform *ESP* charge fitting (Besler et al., 1990) over 10 spherical shells around each atom, with 17 grid points per square Bohr. As *per MCPB.py*’s default (Li and Merz, 2016), the atomic radius of both Fe^2+^ cations was taken to be 1.409 Å. In all DFT calculations, Fe^2+^ centers in both the catalytic and noncatalytic binding sites were modeled in their quintet state, after comparative optimizations of each site with Fe^2+^ in the triplet and singlet state confirmed—at the B3LYP (Lee et al., 1988; Becke, 1993)/6-31G(*d*) level of theory—that the quintet state is the most energetically stable in both cases (data not published). All remaining LH3/PLOD3 residues—including intra-residue bonded parameters for residues in both Fe^2+^-binding sites—were treated with the *ff14SB* forcefield (Maier et al., 2015), whereas Na^+^ countercations were modeled with parameters by Joung and Cheatham (Joung and Cheatham, 2008): these are compatible with the TIP3P model in use for water (Jorgensen et al., 1983).

## Results

### The LH catalytic site does not accommodate competitive inhibitors

The presence of amino acid networks shared by Fe^2+^, 2-OG-dependent dioxygenase enzymes in their catalytic sites provides a general template for structure-based design of potential inhibitors. The analysis of the residues surrounding the catalytic Fe^2+^ and the 2-OG indeed supported the possibility that 2-OG analogs may act as competitive inhibitors of the co-substrate molecule (Rose et al., 2011). In human LH3/PLOD3, the catalytic Fe^2+^ is strongly coordinated by the side chains of His667, Asp669, and His719 of the DSBH fold, and by the 2-OG co-substrate, capped towards the outer solvent by a flexible capping loop defined by residues 590-610 (Figure 2). Using a combination of nano-differential scanning fluorimetry (nanoDSF) and luminescence-based activity assays, we screened a small, focused library of 2-OG analogs (Table 2) searching for compounds capable of inhibiting LH activity. After thorough testing, none of the compounds tested showed binding/folding stabilization in nanoDSF, nor inhibition of enzymatic conversion of 2-OG into succinate. Likewise, a custom-designed library of compounds selected through *in silico* virtual screening of specific candidate binders of the LH3/PLOD3 catalytic site did not provide suitable hints for inhibitors of LH activity using these assays.

**TABLE 2:** List of 2-OG analogs tested. For each compound, the chemical formula of the associated free acid is reported. None of the compounds yielded detectable binding to LH3/PLOD3 or inhibition of the conversion of 2-OG into succinate in presence or absence of acceptor substrate.

Compound	Formula
Formate	CH_2_O₂
Oxalate	C_2_H_2_O_4_
Malonate	C_3_H_4_O_4_
Tartronate	C_3_H_4_O_5_
Mesoxalate	C_3_H_2_O_5_
Aminomalonate	C_3_H_5_NO_4_
Fumarate	C_4_H_4_O_4_
Oxalacetate	C_4_H_4_O_5_
Malate	C_4_H_6_O_5_
Aspartate	C_4_H_7_NO_4_
Tartrate	C_4_H_6_O_6_
Glutamate	C_5_H_9_NO_4_
Glutarate	C_5_H_8_O_4_
Acetonedicarboxylate	C_5_H_6_O_5_
2-hydroxyglutarate	C_5_H_8_O_5_
Adipate	C_6_H_10_O_4_

### A second Fe^2+^ binding site on the capping loop modulates accessibility to the LH catalytic site

Intrigued by the recalcitrance to inhibition of the LH catalytic site by 2-OG analogs, we focused our attention to the distinguishing features displayed by this domain when compared to homologous Fe^2+^, 2-OG-dependent dioxygenases, and in particular to the capping loop and the stable conformation adopted in the presence of excess [Fe^2+^]. This interlocked state may indeed constitute an obstacle when dealing with inhibition of the LH catalytic site, and the relative positioning of the capping loop is crucial for inhibitor accessibility to the active pocket. We decided to perform a thorough investigation of the flexibility of this loop *in silico* and with site-directed mutagenesis *in vitro* to validate the possible significance of the second Fe^2+^ binding site prior to attempting to quantitatively probe the specific metal ion binding to the two distinct LH3/PLOD sites within the LH domain.

To investigate the impact of the second Fe^2+^ on the structural conformation of the capping loop 590-610, the C-terminal LH domain involved in LH/PLOD dimerization underwent all-atom MD simulation in explicit solvent, with and without the second Fe^2+^ ion. In the following, we refer to the simulated system with the second Fe^2+^ as LH3_Fe2_, while the system simulated without it has been named LH3_Fe1_. Three independent replicate simulations were carried out for the two systems, each 1 μs long. In each independent replicate we used identical simulation parameters (see Material and Methods), varying only the initial velocities of atoms via random assignments from a Maxwell distribution consistent with the required temperature. Visual inspection of MD simulations shows that both systems are characterized by minimal atom fluctuations, suggesting that this fragment of the enzyme is stiff and allows for minimal protein motions away from the starting (crystal) structure, except for the gate loop and some protein region at the dimer interface.

**FIGURE 3:**
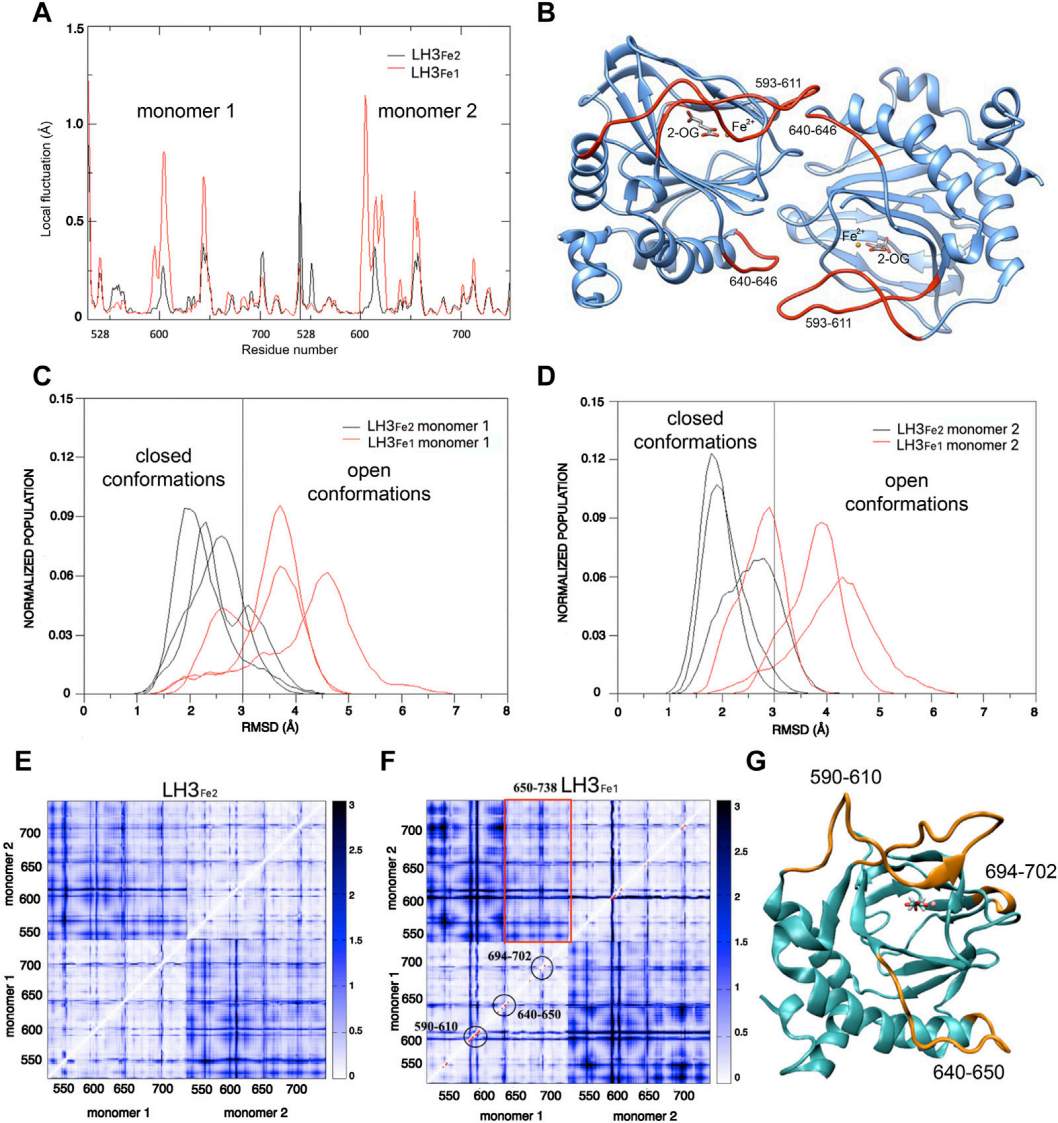
Computational analysis of the dynamic behavior of LH capping loop in modulating accessibility to the LH catalytic site. **(A)** Mean values of the local fluctuations for each residue of the dimerization LH domains of LH3, calculated over the three replicate simulations for LH3_Fe1_ (red profile) and LH3_Fe2_ (black profile) systems. **(B)** Structure of the dimerization LH domains of LH3: residues corresponding to the highest peaks in the local fluctuations profile of LH3_Fe1_ (593-611 of the gate loop and residues 640–646) are colored in red. **(C,D)** RMSD distributions (loop 590–610) of the LH3_Fe2_ (black lines) and LH3_Fe1_ systems, for both LH domains. **(E,F)** Distance Fluctuations analysis. Color-coded DF matrices resulting from the DF analysis for the LH3_Fe2_ **(E)** and LH3_Fe1_ **(F)** systems. Pairs of amino acids that move in a coordinated way, that is with a quasi-rigid motion, are characterized by small DF values (white dots) and vice versa (blue dots). Red dots in the LH3_Fe1_ matrix correspond to residues which show significant difference in DF values compared to the LH3_Fe2_ matrix, according to the F-test. Red rectangle encloses residues 650-738 of one monomer, corresponding to the beta-sheet core of the LH domain, that move in a coordinated way with the other monomer. **(G)** Structure of the LH domain of LH3: residues which show significant differences in protein motion for the two systems are colored in orange.

The first step in the analysis of the dynamics of LH3/PLOD3 C-terminus and its potential variation as a function of the presence/absence of the second Fe^2+^, was the identification of protein regions displaying higher levels of local flexibility during dynamics, through the computation of the local fluctuations (LF). This parameter detects the flexibility of a given residue with respect to the neighboring amino acids; the comparison of this calculation for the two systems (i.e., LH3_Fe1_ and LH3_Fe2_), allows to extrapolate variations in protein regions more affected by the presence/absence of the second Fe^2+^. LF is calculated for residue *i*, as the mean of the variances of the distance d_*ij*_ of the C_α_ (i) and C_α_ (j) atoms, for j = i-2, i-2, i+1, i+2. In the LH3_Fe1_ system residues 593-611 of the gate loop of both monomers displayed higher flexibility as compared to the LH3_Fe2_ system (Figures 3A,B). The peak at residues 640-646, constituted by residues of one monomer which contact the gate loop of the other monomer, is more defined in the LH3_Fe1_ system (Figures 3A,B). To quantify the observed differences in the dynamics of the enzyme during MD in the two simulated systems, we calculate the root-mean-square deviation (RMSD) distributions of MD trajectories, after optimal rigid body superposition of backbone atoms of each snapshot with the corresponding atoms of the crystal structure, where the gate loop is in the closed conformation.

The RMSD distributions of the loop 590-610 in the two monomers of the single trajectories are reported in Figures 3C,D, showing the different behavior of the two systems and the variations in the single trajectories. A threshold of 3 Å was used to distinguish the closed from the open conformation, based on visual inspection of MD simulations and superposition of snapshots representing the two states. These plots show that the gate loop is stabilized in the closed conformation in the LH3_Fe2_, while the absence of second Fe^2+^ impacts the stability of the loop, shifting the population of LH3_Fe1_ towards the open conformation, even though both systems can visit the two states. To understand the impact of the second Fe^2+^ on the internal dynamics of LH3/PLOD3, we performed the distance fluctuation analysis on the meta trajectories of the two systems, obtained concatenating the three MD replicas of each system. The DF parameter, which is the variance of the inter-residue distance d_ij_, was calculated for any pair of residues during the trajectory (see Material and Methods). The resulting DF matrices calculated for LH3_Fe1_ and LH3_Fe2_ shown in Figures 3E,F, report on the fluctuation of the inter-residue distance in the corresponding residue pairs and matrix regions, describing the intrinsic flexibility and coordination of the LH domains. Relatively low DF values identify protein regions that move together, in coordination. The comparison of these matrices can be used to evaluate possible changes of the internal dynamics and coordination due to the presence/absence of the second Fe^2+^.

Overall, the matrices for both systems turned out to be similar, with largely overlapping patterns of small and large fluctuations of inter-residue distances. In both systems, the internal dynamics of the two domains is characterized by small atomic fluctuations, confirming that protein structure is stiff. Indeed, the highest DF parameters in both systems correspond to protein regions that do not adopt well-ordered 3D structures, which are intrinsically more flexible. Generally, the inter-domain motion is characterized by higher fluctuations than the internal domain motion. In particular, in the LH3_Fe1_ system, residues 650-738 of one monomer, corresponding to the beta-sheet core, move in a more coordinated way with the other monomer than the rest of the protein (Figure 3F).

The significance of the differences observed between the two DF matrices was evaluated with a statistical analysis based on F-test. We used LH3_Fe2_ as a reference state for comparing the two matrices. Red dots in the LH3_Fe1_ system (Figure 3F) correspond to residues which show significant difference in DF values in respect to the corresponding DF values in LH3_Fe2_, according to this test. This analysis highlights that relevant differences in protein motion for the two systems concern residues of the gate loop 590-610 and residues 640-650 forming the loop that connects the alpha helix 618-639 with the beta-sheet core of the protein. This loop partially forms the dimerization interface of the two domains, as well as residues 694-702, which displays higher DF values in the LH3_Fe1_ system (Figures 3F,G).

**FIGURE 4:**
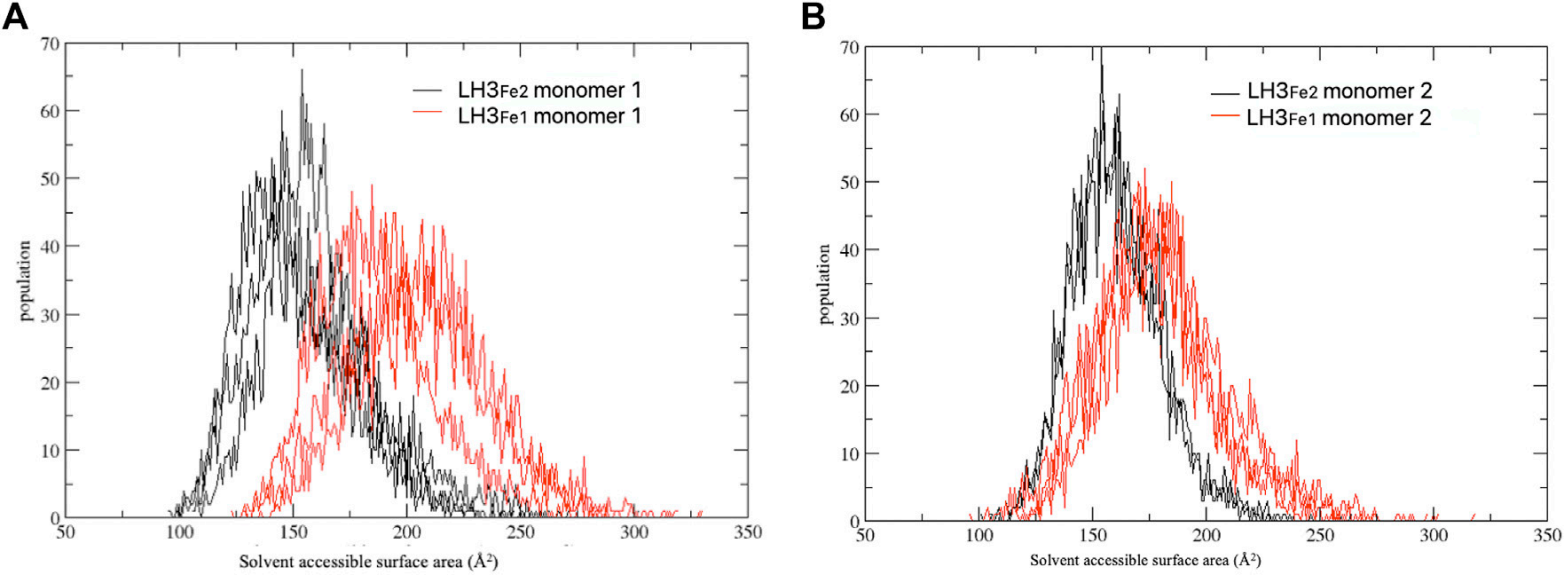
Distribution of the solvent accessible surface area calculated for the three replicate simulations. The simulations have been carried out separately for each computed system (LH3_Fe1_ in red, LH3_Fe2_ in black) and monomer **(A,B)**.

To further investigate the role of the relative positioning of the capping loop for the accessibility to the active pocket depending on the second Fe^2+^ ion, we computed the part of the van der Waals surface of residues forming the catalytic site (protein residues within 6 Å of 2-OG and Fe^2+^) that are accessible to solvent, that is the solvent accessible surface area, across each frame of MD trajectories, using the *VMD* program (Humphrey et al., 1996). This analysis gives a measure of the capability of the loop in regulating the access of the substrate to the catalytic site, depending on the presence of the second Fe2+. Figure 4 shows the distribution of the solvent accessible surface area calculated for the three replicas, for each system and monomer. In both monomers the distributions of the LH3_Fe1_ system are shifted towards slightly higher values compared to the LH3_Fe2_, suggesting that the presence of the Fe^2+^ has an impact on the access to the active site by modulating the opening/closure of the loop, even though both systems can visit the two distinct states, in agreement with the RMSD analysis discussed above.

To inspect the effect of the second Fe^2+^ on the dynamics of the capping loop at a finer level, we evaluated the persistence over the simulation time of the native and new interactions established by this loop with the rest of the protein. The results of this analysis show that most of the native contacts are maintained during the simulation time in both systems (Supplementary Figure S1A,B), while in LH3_Fe1_ some residues of the loop tend to weaken some of these interactions compared to LH3_Fe2_. As for new stable contacts, we observed that in LH3_Fe1_ the loop can’t form new interactions with the rest of the protein due to its higher mobility, while in the LH3_Fe2_ the loop establishes new steady interactions with residues 643-648 of the other monomer (Supplementary Figure S1C).

As mentioned above, in the crystal structure of the LH domain obtained in the presence of excess [Fe^2+^], residue Arg599 belonging to the loop 590-610 forms a salt bridge with the 2-OG co-substrate, yielding a conformation that mimics the collagen lysine substrate (Scietti et al., 2018) (Figures 2C,D). To check the impact of the presence/absence of the second Fe^2+^ on the stabilization of this bond, we tracked the hydrogen bonds between Arg599 and 2-OG co-substrate over the course of the trajectories. This analysis showed that in most of the conformations visited during the dynamics, Arg599 forms a salt-bridge with 2-OG in both systems, with a in slight prevalence in LH3_Fe2_ (Supplementary Figure S1D–F). Taken together, the results from the *in silico* analysis corroborate the observation that the second Fe^2+^ constrains the LH domain in a tightly interlocked conformation with reduced conformational motility in proximity to the enzyme’s catalytic site.

**FIGURE 5:**
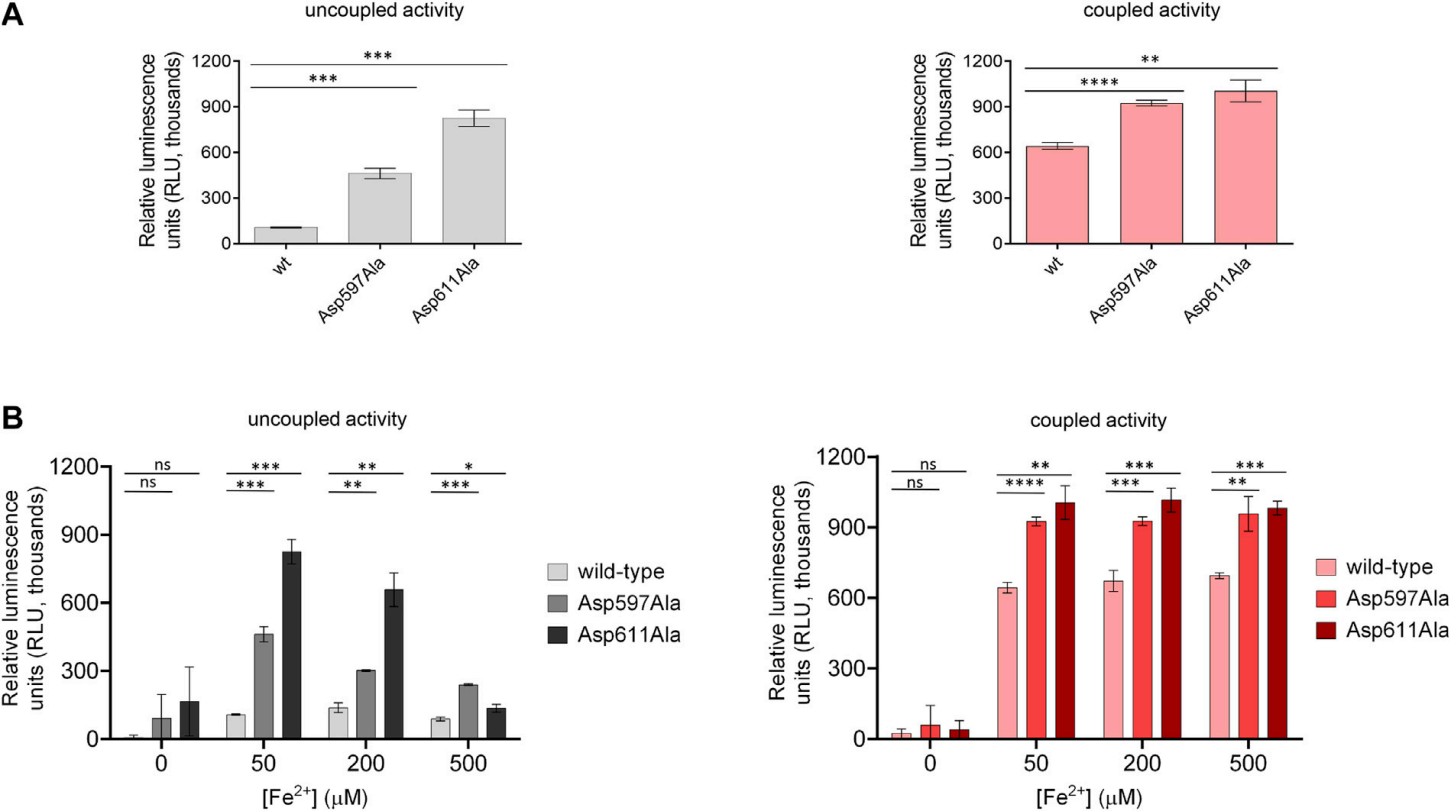
Comparison of enzymatic activity of LH3/PLOD3 wild-type and mutants affecting the second Fe^2+^ binding site. **(A)** Luminescence-based uncoupled (i.e., with no acceptor substrate, left) and coupled (i.e., using gelatin as acceptor substrate, right) enzymatic activity assays detecting succinate formation. Error bars represent standard deviations from average of triplicate independent experiments. Statistical evaluations based on pair sample comparisons between luminescence values for wild-type LH3 and mutants using Student’s *t*-test. ns, non-significant; *, *p*-value<0.05; **, *p*-value<0.01; ***, *p*-value<0.001; ****, *p*-value < 0.0001. **(B)** Evaluation of the uncoupled (left) and coupled (right) enzymatic activities [as in panel **(A)**] in presence of increasing concentrations of Fe^2+^. Error bars and statistical analyses as in panel **(A)**.

To better examine the impact of the second Fe^2+^-binding site on enzymatic activity and substrate accessibility, we generated the Asp597Ala and Asp611Ala variants of human LH3/PLOD3 using site-directed mutagenesis. In particular, we focused on Asp597 as a critical capping loop residue involved in the coordination of the second Fe^2+^, whereas Asp611 could represent its counterpart as central to the Fe^2+^ coordination platform on the surface of the LH domain (Figures 2C,D). Both mutants could be expressed and purified to homogeneity, and showed yields, folding stability and oligomerization states comparable to wild-type LH3/PLOD3 (Supplementary Figure S2). We probed their Fe^2+^ binding affinity using Temperature-Related Intensity Change (TRIC) and found that both mutants showed 4-times lower affinity compared to wild-type LH3/PLOD3 (Supplementary Figure S3). We were not surprised of the weak binding observed: the catalytic Fe^2+^ is known to have structural roles within the LH domain and cannot be removed to probe its binding (Guo et al., 2018; Scietti et al., 2018), therefore we assumed that the binding data collected exclusively refer to possible additional Fe^2+^ binding sites. When tested for their enzymatic activity in the presence of acceptor substrates such as gelatin (i.e., coupled activity), the mutants showed slightly higher (i.e., 1.5X) activity than their wild-type counterpart (Figure 5A, right). Nevertheless, we consistently observed a much higher (i.e., 3-5X) degree of 2-OG conversion into succinate without the need of an acceptor substrate (i.e., uncoupled activity, Figure 5A, left). Prompted by this observation, we decided to investigate the modulatory effect of [Fe^2+^] on both uncoupled and coupled enzymatic activities of these mutants. We found that the uncoupled activities of both LH3/PLOD3 Asp597Ala and Asp611Ala mutants were less inhibited by high [Fe^2+^] compared to wild-type LH3/PLOD3 (Figure 5B, left), whereas the modulation of the coupled enzymatic activities was almost unaffected by the presence of the point mutations (Figure 5B, right). Based on the combined results obtained from the MD simulations and mutagenesis, we reasoned that binding of a second metal ion on the surface of the LH domain induces a conformationally stable, self-inhibited state which in turn reduces the ability of LH/PLOD enzymes to process 2-OG into succinate via uncoupled activity.

### Fe^2+^ chelating agents produce unexpected effects on LH3/PLOD3 enzymatic activity

**FIGURE 6:**
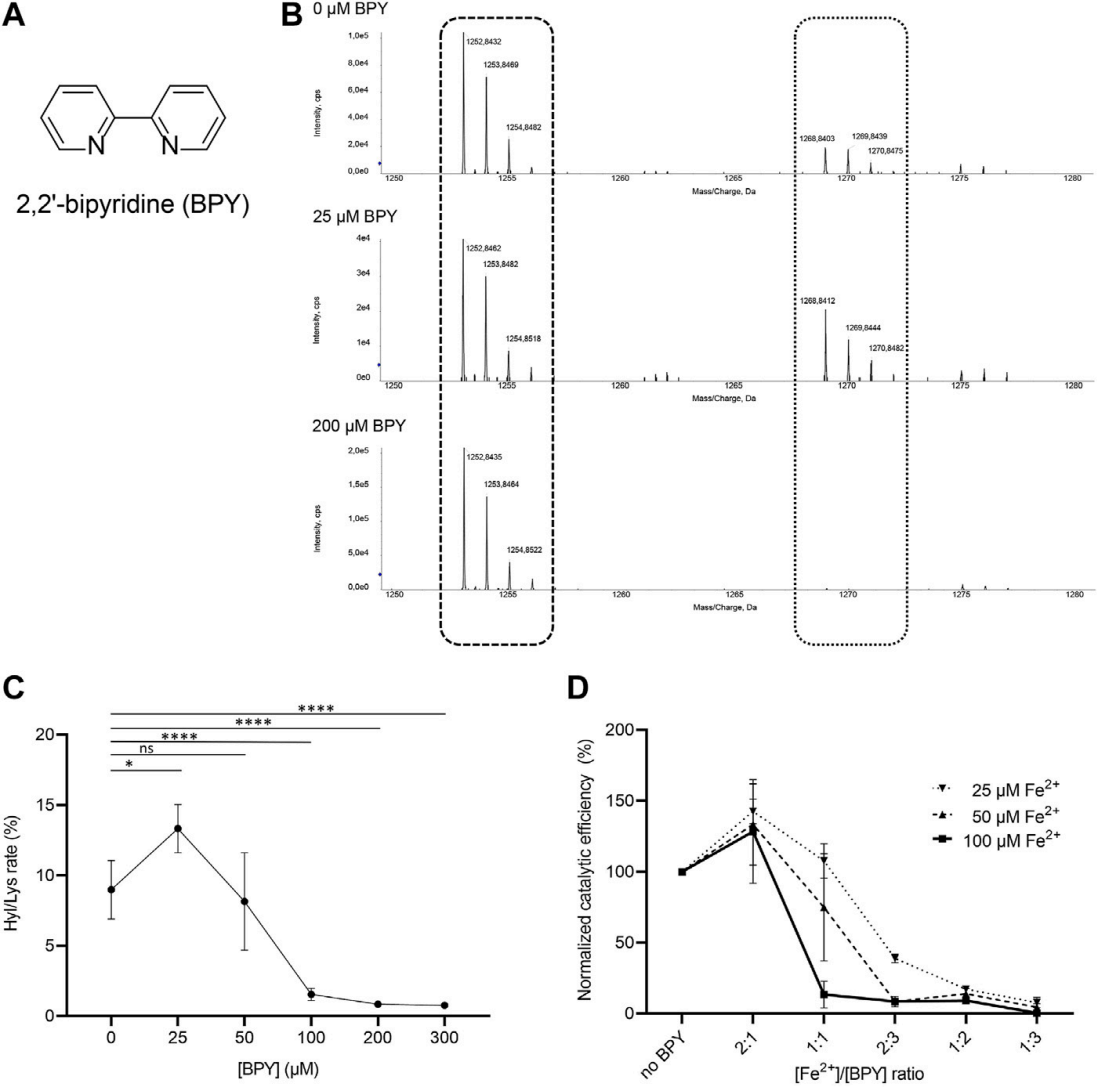
Evaluation of the effect of 2,2’-bipyridil (BPY) on LH3/PLOD3 enzymatic activity. **(A)** Chemical structure of BPY. **(B)** Detection of Hyl formation using direct mass spectrometry (MS) assays, and evaluation of the effect of BPY on Lys-to-Hyl conversion. The scheme shows the comparison between three MS spectra, showing the peaks consistently detected for unmodified Lys in the synthetic peptides (dashed box) and the presence of additional peaks (dotted box) indicating Hyl formation. **(C)** Results of MS analysis of LH3/PLOD3 enzymatic activity as a function of BPY concentration. The plot shows the presence of an unexpected increase of enzymatic activity at low BPY concentrations, followed by a drop consistent with inhibition due to sequestration of catalytic Fe^2+^. Error bars represent standard deviations from average of triplicate independent experiments. Statistical evaluations based on pair sample comparisons between data points collected in absence or in presence of [Fe^2+^] using Student’s *t*-test. ns, non-significant; *, *p*-value<0.05; **, *p*-value<0.01; ***, *p*-value<0.001; ****, *p*-value < 0.0001. **(D)** Evaluation of LH3/PLOD3 enzymatic activity as a function of varying [BPY]/[Fe^2+^] ratios. The analysis shows that the enzymatic activity increase, already observed at low BPY concentration, depends on the residual Fe^2+^ concentration available for catalysis. Error bars represent standard deviations from average of triplicate independent experiments. Statistical analyses conducted by comparing data points collected at the same [Fe^2+^]/[BPY] ratio using one-way ANOVA analysis highlight non-significant differences for experiments performed using different concentrations of Fe^2+^ in the assay. Additional statistical evaluations for each data point are provided in Supplementary Figure S3.

Given the unique presence of two distinct Fe^2+^ binding sites in close proximity, but with opposite effects on enzymatic activity, we wondered whether small-molecule inhibitors acting through metal ion chelation could efficiently modulate substrate processing in LH/PLOD enzymes. To date, despite large scale screenings identified potential hits (Devkota et al., 2019), specific inhibitors of the lysyl hydroxylase activity are missing. The only known inhibitor of LH/PLOD enzymes is the 2,2’-bipyridine (BPY), a non-specific inhibitor that act as chelating agent. BPY chemical structure is characterized by two pyridyl rings, heterocyclic chemical moieties containing nitrogens (Figure 6A). Although the mechanism of action of BPY on LH/PLOD enzymes is not well characterized, being a metal chelator, it likely acts on the Fe^2+^ ion present in the lysyl hydroxylase domain (Ikeda et al., 1994; Rose et al., 2011; Vasta and Raines, 2015; Jover et al., 2018). We therefore decided to explore the actual impact of BPY inhibition on human LH3/PLOD3 enzymatic activity. Firstly, we attempted to evaluate concentration-dependent inhibition using luminescence-based assays, but we realized that usage of BPY was not compatible with the assay setup, likely due to inhibition of the Mg^2+^-dependent luciferase reaction by the reagent. We therefore focused on a strategy to directly investigate hydroxylysine formation on synthetic collagen peptides using mass spectrometry (MS) by evaluating the relative ratios between non-hydroxylated and hydroxylated lysine side chains (Figure 6B). Using this method, we could perform accurate quantitation of the dose-dependent effects of BPY on LH3/PLOD3 enzymatic activity.

In absence of BPY, collagen peptide substrate processing was consistent with previously reported data (Scietti et al., 2018). Given a BPY:Fe^2+^ chelation stoichiometry of 3:1, and a half-maximal BPY concentration required to form complexes with 20 μM Fe^2+^(Fe_20_–EC_50_) experimentally determined in about 40 μM (Vasta and Raines, 2015), we would have expected an initial reduction of LH activity at a [Fe^2 +^]/[BPY] ratio of 2:1. Surprisingly, assays performed with concentrations of BPY up to 25 μM unexpectedly revealed increasing Hyl/Lys ratios, suggesting enhancement of enzymatic activity, whereas only higher BPY concentrations caused the expected dramatic decay in substrate processing (Figures 6B,C). Considering that standard assays are carried out by supplementing a fixed concentration of Fe^2+^ (i.e., 50 μM), we wondered whether the activity boost observed at relatively low BPY concentrations could be associated to Fe^2+^ sequestration from the non-catalytic site on capping loop, whereas inhibition could be caused by chelation of the catalytic Fe^2+^.

We therefore carried out additional assays, in which we simultaneously varied [Fe^2+^] and [BPY]. Our results (Figure 6D, Supplementary Figure S4) showed that the LH3/PLOD3 catalytic activity always reaches its maximum at [Fe^2+^]/[BPY] ratio of 2:1, supporting a fine balance between sequestration of excess inhibitory metal ions bound to the capping loop and inhibition through chelation of Fe^2+^ in the catalytic site. These results suggest that usage of BPY as a lead compound for the development of inhibitors of LH/PLOD enzymatic activity demands particular care, as unexpected concentration-dependent opposite effects may be produced through release of the self-inhibitory, Fe^2+^-dependent capping loop.

## Discussion

Human LH/PLOD enzymes are becoming a hot topic in cancer research, due to their involvement in fibrotic conversion of collagens in the tumor microenvironment and the association to higher risk of metastasis. As cancer metastatization has been clearly correlated to excess Lys-Hyl enzymatic conversion, development of highly specific LH/PLOD inhibitors is desirable. With the release of the molecular structure of full-length human LH3/PLOD3, the challenge of securing detailed atomic structures for at least one human LH/PLOD isoform has been overcome (Scietti et al., 2018), however as of yet no LH/PLOD inhibitors are available. In this work, we carried out a small molecule screening aiming at finding hits to be used for the development of LH/PLOD inhibitors. To achieve this goal, we used structural and mechanistic insights from related Fe^2+^, 2-OG-dependent dioxygenases, and found that the challenge of developing specific LH/PLOD inhibitors may present additional obstacles for which extra care is needed.

Despite the high resemblance of the LH3/PLOD3 catalytic pocket with structurally-related Fe^2+^, 2-OG dioxygenases that could be inhibited by 2-OG analogs (Rose et al., 2011), our initial campaign focused on development of small-molecule inhibitors based on their ability to compete with 2-OG in the LH catalytic site were not successful. Likewise, dedicated libraries of compounds developed *in silico* based on high-resolution structural templates of the enzyme cavity did not provide any useful leads towards LH/PLOD inhibition. Using MD simulations, we could interpret the systematic recalcitrance to inhibition with limited accessibility to the catalytic site, caused by a very stable, self-inhibited state generated by specific conformations adopted by the capping loop in the presence of excess [Fe^2+^]. Previous structural studies demonstrated that the capping loop folding was strictly dependent on the iron coordination by four residues, three of which laying on the surface of the LH domain (i.e., His595, Asp611, His613), and one (Asp597) being part of the capping loop. By mutagenizing either Asp611 or Asp597 to alanine, we could observe a decrease in the binding affinity for non-catalytic Fe^2+^ and a much-increased uncoupled conversion of 2-OG into succinate which can be explained with improved accessibility to the catalytic site, supporting the physiological role of the Fe^2+^-dependent self-inhibited conformation adopted by the capping loop.

Taken together, these results highlight the extremely delicate balance of Fe^2+^ concentration needed in LH/PLOD enzymes to enable their function: while too little [Fe^2+^] hampers catalysis due to lack of an essential component in the catalytic site, even a little excess can instead interlock the LH domain into a self-inhibited state. This behavior is unique for LH/PLODs and different from related Fe^2+^, 2-OG dioxygenases, and is likely responsible for the differential responses observed during treatment with Fe^2+^ chelators. Indeed, when testing BPY as candidate inhibitor of LH activity, we found that at low concentrations this compound is capable of enhancing the enzymatic activity rather than blocking it. We interpreted this boost in enzymatic activity with sequestration of the Fe^2+^ bound on the LH domain surface trapping the capping loop, and we could demonstrate that such effect depends on the ratio between [Fe^2+^] and [BPY].

Collectively, the results obtained provide interesting perspectives regarding the mechanisms of substrate processing by LH/PLOD enzymes, as well as guidance for future inhibitor design. The strong stability of the Fe^2+^-induced conformation adopted by the capping loop, together with the self-inhibited state obtained through substrate mimicry by LH3/PLOD3 Arg599 implies that physiological substrate processing may depend on the release of the second Fe^2+^ interlock through long-range interactions on the surface of the LH domain. Given the extended conformation of collagen polypeptide substrates, this is a likely option and demands further investigation, in particular considering the possible roles that metal ions bound to collagen substrates may have upon/during post-translational modification of lysines, but also after the processed collagen has detached from the LH/PLOD enzymes. As for inhibitor design, the presence of this metal ion-dependent self-inhibited state represents an additional challenge, which may require the development of synergistic strategies acting simultaneously on the release of the capping loop and on the competition with either 2-OG in the catalytic site, or the Lys side chain subjected to hydroxylation.

## Data Availability

The original contributions presented in the study are included in the article/Supplementary Material, further inquiries can be directed to the corresponding authors.
