# Supplementary material for: A Fe2+-dependent self-inhibited state influences the druggability of human collagen lysyl hydroxylase (LH/PLOD) enzymes
Source: Front Mol Biosci. 2022 Aug 25;9:876352. doi: 10.3389/fmolb.2022.876352 (PMC9453210; doi:10.3389/fmolb.2022.876352)
Supplement: Supplementary file 1 [file DataSheet1.pdf]

Supplementary Information for:

A Fe^{2+}-dependent self-inhibited state influences the druggability of human collagen lysyl hydroxylase (LH/PLOD) enzymes

Luigi Scietti, Elisabetta Moroni, Daiana Mattoteia *et al.*

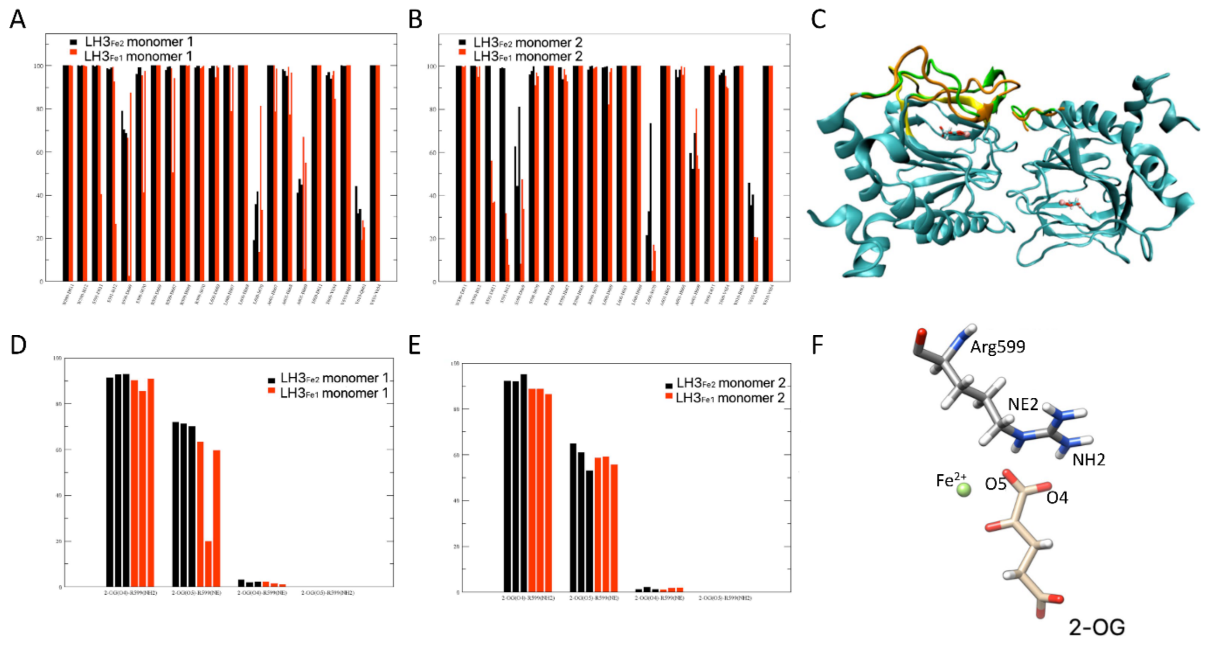

Supplementary Figure 1. Results from computational investigations. (A-B) Persistence of the native contacts of the loop 590-610 obtained from MD simulations, calculated for the three replicate simulations, for each system and monomer. The persistence of a contact is the number of frames in which this contact is present, divided by the total number of frames of MD simulation. A contact is considered as native if it is present in the crystal structure. A contact is considered as "present" when the distance of one of the C_α atoms of residues of the loop and one of the C_α atoms of residues not belonging to the loop is lower than 7.0 Å. (C) Snapshot from MD simulation of **LH3**$_{\mathbf{Fe1}}$ (cyan) superimposed to the crystal structure (orange). Residue of the loop 590-610 and residues 643-648 which form stable contacts with this loop during the dynamics, are colored in green, while the corresponding residues in the crystal structure are colored in orange. Yellow residues correspond to the protein portion where the native contacts are less stable in the **LH3**$_{\mathbf{Fe1}}$ system. (D-E) Persistence of hydrogen bonds between Arg599 and 2-OG co-substrate in both monomers over the course of the MD trajectories, calculate for the three replicate simulations. Hydrogen bonds are determined using a geometric criteria: the donor to acceptor heavy atom distance (distance of 3.0 Å) and the donor-hydrogen-acceptor angle (angle cutoff of 135°). (F) Atom names used to define donors and acceptors for Arg599 and 2-OG.

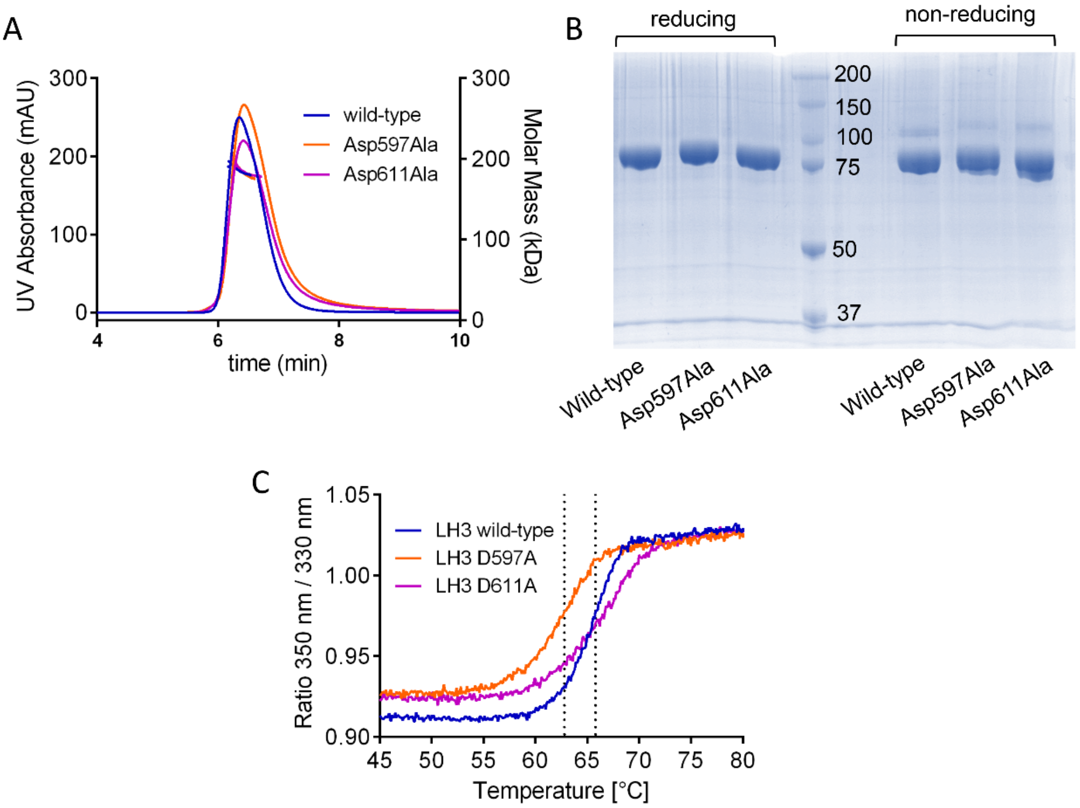

Supplementary Figure 2. Biochemical characterization of LH3/PLOD3 mutants and comparison with wild-type enzyme. (A) Comparative size exclusion chromatography coupled to multi-angle light scattering (SEC-MALS) analysis. The dots indicate the computed molar mass associated to each sample. (B) SDS-PAGE analysis. Shown are the results obtained in reducing (left) and non-reducing (right) conditions. (C) Differential scanning fluorimetry analysis. The dashed lines indicate the temperature range incorporating the calculated unfolding temperature values for all curves.

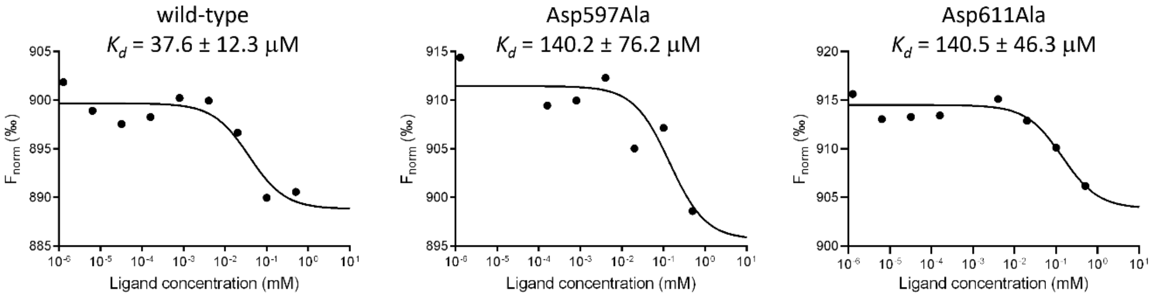

Supplementary Figure 3. Analysis of Fe^{2+} binding in wild-type and mutant LH3. Normalized fluorescence values (F_{norm}) extrapolated from TRIC curves obtained from the Dianthus NT.23 (NanoTemper Technologies) were plotted as as a function of Fe^{2+} concentration and used to compute the K_d values reported with the associated standard deviations as obtained from the binding affinity analysis performed using the using the DI.screening analysis software (NanoTemper Technologies).

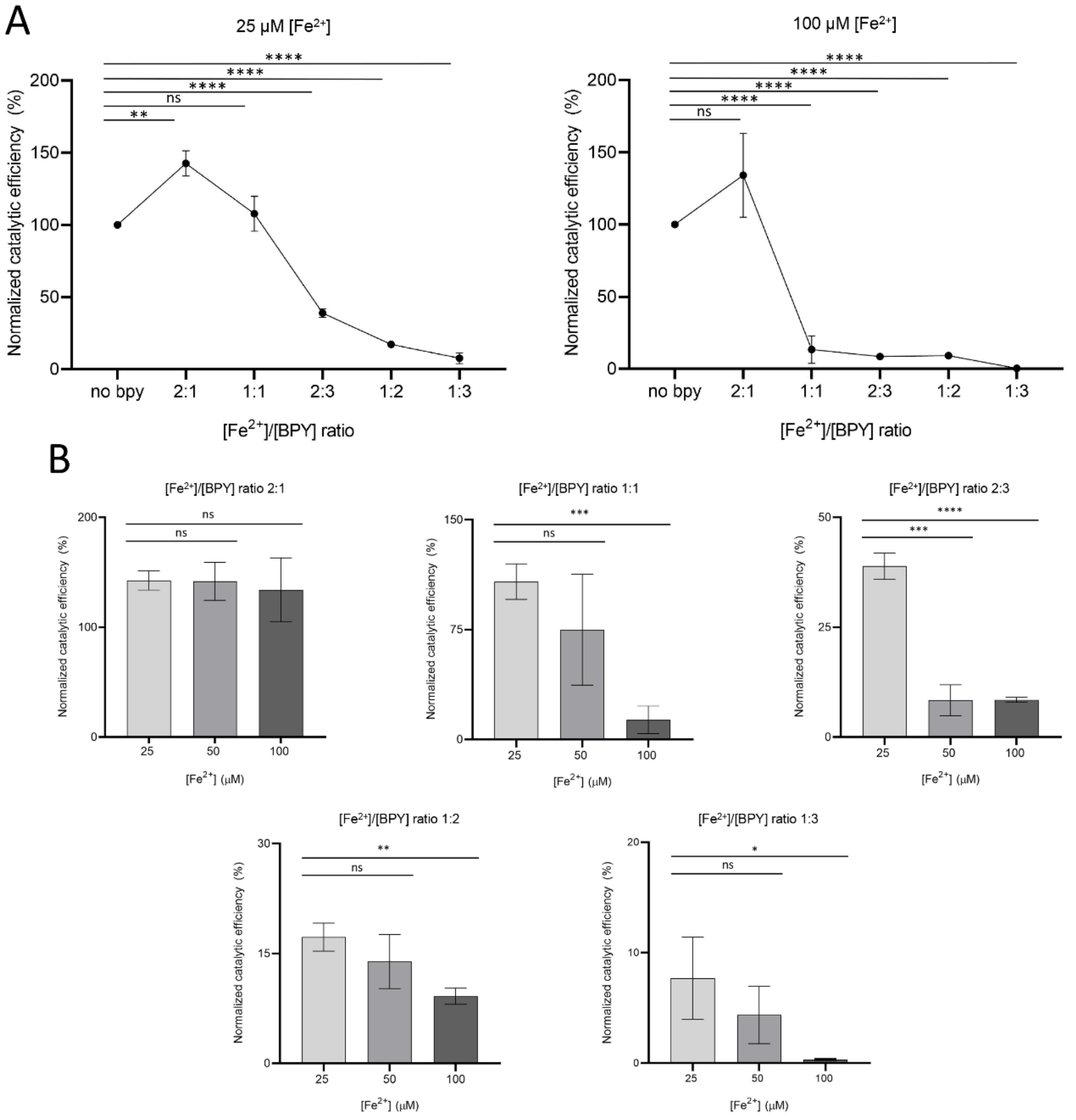

Supplementary Figure 4. Detailed statistical evaluation of the data shown in Figure 6. (A) Results of the MS analysis of LH3/PLOD3 enzymatic activity as a function of the [Fe^{2+}]/[BPY] ratio in assays performed using 25 µM Fe^{2+} (left) or 100 µM Fe^{2+} (right). Error bars represent standard deviations from average of triplicate independent experiments. Statistical evaluations based on pair sample comparisons between data points collected in absence or in presence of [Fe^{2+}] using Student's t-test. ns, non-significant; *, P-value<0.05; **, P-value<0.01; ***, P-value<0.001; ****, P-value < 0.0001. (B) Pairwise statistical evaluation of each individual [Fe^{2+}]/[BPY] ratio as a function of different [Fe^{2+}] used in the assay.
